# Keto-Adaptation and Endurance Exercise Capacity, Fatigue Recovery, and Exercise-Induced Muscle and Organ Damage Prevention: A Narrative Review

**DOI:** 10.3390/sports7020040

**Published:** 2019-02-13

**Authors:** Sihui Ma, Katsuhiko Suzuki

**Affiliations:** 1 Graduate School of Sport Sciences, Waseda University, 2-579-15 Mikajima, Tokorozawa 359-1192, Japan; masihui@toki.waseda.jp; 2 Faculty of Sport Sciences, Waseda University, 2-579-15 Mikajima, Tokorozawa 359-1192, Japan

**Keywords:** ketogenic diet, keto-adaptation, fatigue, muscle damage, organ damage

## Abstract

A ketogenic diet (KD) could induce nutritional ketosis. Over time, the body will acclimate to use ketone bodies as a primary fuel to achieve keto-adaptation. Keto-adaptation may provide a consistent and fast energy supply, thus improving exercise performance and capacity. With its anti-inflammatory and anti-oxidative properties, a KD may contribute to muscle health, thus preventing exercise-induced fatigue and damage. Given the solid basis of its potential to improve exercise capacity, numerous investigations into KD and exercise have been carried out in recent years. This narrative review aims to summarize recent research about the potential of a KD as a nutritional approach during endurance exercise, focusing on endurance capacity, recovery from fatigue, and the prevention of exhaustive exercise-induced muscle and organ damage.

## 1. Introduction

Our metabolic system is remarkably flexible in its ability to use a variety of dietary macronutrients as fuels. Traditionally, carbohydrate-centered diets have been recommended for sports [1,2,3,4,5]. Carbohydrate-loading is one of the main nutritional strategies to improve exercise performance before crucial events [6,7,8,9,10]. However, for excellent athletes competing in endurance sports, this strategy may lead to an awkward dilemma. The capacity for the human body to store carbohydrates is limited, which is 5 g glucose in blood circulation and ~100 g or ~500 g glycogen in skeletal muscle or liver. A person of average weight may store 10 kg fat in the body, which makes the person capable of completing >30 marathon races if the stored fat is effectively utilized [11,12,13,14]. However, the abundant carbohydrates in traditional high-fat diets may restrict us from utilizing fat [15,16,17,18,19,20]. Therefore, whether we can find an effective way to enhance fat utilization is an important question.

A ketogenic diet (KD) involves using fat, a high-density substrate, as the main source in daily calorie intake while restricting carbohydrate intake [21,22]. In this way, the liver is forced to produce and release ketone bodies into the circulation [23,24,25,26]. This phenomenon is called nutritional ketosis [27,28,29]. Over time, the body will acclimate to using ketone bodies as a primary fuel, which is called keto-adaptation, an element of fat-adaptation [30,31,32]. Glucose oxidation requires 11 steps to produce energy, whereas fat and ketone bodies can quickly provide energy in only three steps [33,34]. In any case, the capacity for the body to reserve ketone bodies and fat is much stronger. Compared to glucose, ketone bodies are more energy-intensive, while a ketone body-centered metabolism has the potential to provide a consistent, fast energy supply [35]. Despite the potential to increase exercise performance and capacity, a KD may also contribute to muscle health by anti-inflammatory and anti-oxidative properties, thus preventing exercise-induced fatigue and muscle and organ damage [35,36,37,38,39,40,41,42,43,44]. Compared to glucose oxidation, fat-centered oxidation involves producing less reactive oxygen species during the process. Excessive free radicals and chronic inflammation are harmful to mitochondria, muscular cells, and whole-body health [45]. Long-term KD administration is linked with reduced inflammatory mediators by down-regulating NACHT, LRR, and PYD domain-containing protein 3 (NLRP3) inflammasome expression and reducing the generation of isoprostanes [46,47]. It is also reported that a high-fat diet could contribute to mitochondrial biogenesis and reduce mitochondrial autophagy, thus contributing to a rich mitochondrial reservoir in the muscle, boosting exercise performance, and contributing to athletes’ wellbeing [48,49]. 

Given the solid basis of its potential to improve exercise capacity, numerous investigations on KD and exercise have been carried out in recent years. This review aims to summarize the recent literature (mainly articles published in the past three years) about the potential of KDs as a nutritional approach during endurance exercise, in a narrative way. We searched MedLine extensively with the Medical Subject Headings (MeSH term) diet, ketogenic, ketone body, ketosis, and related keywords to access related articles. The main focus of this article is on endurance capacity, fatigue recovery, and the multi-faceted approach to prevent exhaustive exercise-induced muscle and organ damage.

## 2. KD, Keto-Adaptation, and Endurance Exercise Capacity

In interesting research conducted in keto-adapted ultra-endurance runners, keto-adaptation promoted higher peak fat oxidation (2.3-fold higher compared to un-adapted athletes, *n* = 10) [50]. This result has been attributed to increased fat oxidation capacity. In 2016, an article from *Cell Metabolism* reported that exhaustive cycling performance was improved by nutritional ketosis [51]. In 2019, McKay and colleagues reported that in keto-adapted elite race walkers, after a standardized 19–25 km race walk, IL-6, which is the myokine that may induce intense lipolysis, was measured. Subjects that adhered to a low-carbohydrate high-fat diet were significantly higher, exhibiting a potential performance enhancement in endurance capacity. However, as the authors concluded in this paper, the circulating IL-6 might be harmful inflammatory cytokines and may cause side effects [52]. This might be a limitation of the human study, and a well-designed in vivo or in vitro experiment may validate this result. Parry and colleagues reported that in an experiment that lasted for 762 days, muscle mitochondrial volume was increased by a KD (citrate synthase in gastrocnemius, *p* < 0.05 compared with the control, *n* = 8) [53,54]. Shimizu et al. reported that a 12-week KD combined with daily treadmill exercise induced higher gene expression in markers of fatty acid oxidation, as compared with the control diet combined with exercise (*n* = 6) [55]. We also reported that ketolytic metabolism and lipolytic metabolism were re-modeled by an eight-week KD in mice, therefore enhancing their endurance [35,36,37,38]. These results explain the partial mechanisms by which keto-adaption showed great potential in improving endurance exercise capacity.

However, on the contrary, Zinn et al. reported in a pilot study that in New Zealand endurance athletes, KD increased benefit in body composition and wellbeing, but failed to enhance endurance capacity [56]. The mean age of those subjects was 51.2 years old; thus, the generality of this study to younger athletes should be considered. Meanwhile, a low-carbohydrate, high-fat diet impaired exercise economy and performance after intensified training in a group of elite race walkers [57]. In an animal model, however, eight weeks of KD significantly enhanced the endurance capacity of C57/BL6 mice (*n* = 8) [35,36,37]. In this study, a correlation existed between body weight and running time until exhaustion, with mice on heavier a KD running longer. This was attributed to keto-adaptation [35]. Since there was an inter-individual difference, the subjects possessing higher metabolic flexibility may prefer KD and reflect the weight change. After a two-month KD, the average weight of KD mice decreased by 30% compared with normal diet-fed mice [36]. 

## 3. Keto-Adaptation, Fatigue Prevention, and Recovery

Glycogen depletion, lactate accumulation, and oxidative stress are considered the main factors promoting exercise-induced fatigue. While lactate may not be a reason causing fatigue, a higher removal rate of lactic acid is usually employed as a post-exercise fatigue indicator. In 2014, Zajac and colleagues reported that a one-month KD improved the lactate threshold in off-road cyclists [50]. In the article published in *Cell Metabolism*, nutritional ketosis was induced. Lactate concentrations were significantly lower with KD administration, resulting in a 50% reduction in lactate concentrations 30 min after exercise commencement compared to the non-KD group [51]. Carr et al. reported that compared to high-carbohydrate groups, low-carbohydrate KD ingestion contributed to a lower lactate accumulation post-endurance exercise (*n* = 8) [58]. In an animal study, after exhaustive exercise, muscle lactate was much lower in keto-adapted mice. After 24 h of rest, plasma lactate dropped more quickly in the keto-adapted subjects, showing that keto-adaptation has the potential to prevent fatigue or boost recovery after subjects reach fatigue [37]. 

In the study conducted in keto-adapted ultra-endurance athletes, after a 3-h submaximal exercise, muscle glycogen decreased in both KD-adapted athletes and un-adapted athletes, with no difference between them [59]. In another study, keto-adaptation contributed to a slower glycogen drop during 1 h of submaximal exercise [51]. These results imply the metabolic flexibility of muscle glycogen regulation via gluconeogenesis and the conservation of glycogen, thus potentially contributing to the prevention of fatigue.

In a 762-day experiment conducted in sedentary rats, either muscle, liver, or brain superoxide dismutase (SOD) 1 and 2, catalase, glutathione peroxidase (GPX), and 4-hydroxynonenal (4-HNE) conjugated protein were measured at the end, though no significance was observed [53]. In a mouse model involving exhaustive exercise, the hepatic protein carbonyl level was lower in the keto-adapted mice, whereas the same marker was significantly higher in the muscle tissue [37]. However, oxidative stress plays a subtle role in muscle hypertrophy; thus, reservations should be held on this issue. Evidence surrounding keto-adaptation and oxidative stress are limited; thus, future studies in this field are required. 

On the other hand, it is also reported that a KD may induce fatigue perception, as a direct correlation between blood ketone bodies and fatigue was reported [60]. However, this study was conducted in overweight subjects, and the results may not relate to athletic groups. To summarize, though some negative results of keto-adaptation are reported, a solid basis and abundant evidence about the potential of KD on fatigue prevention and recovery makes it necessary to validate the efficacy of KD in future studies. 

## 4. Keto-Adaption and Exercise-Induced Muscle/Organ Damage 

Some scholars and researchers question whether a KD may contribute to weight loss, which may induce decreased muscle volume. However, in a randomized control trial, gymnasts consuming a one-month KD while receiving the same training did not lose muscle; instead, they experienced a non-significant increase of muscle mass (pre, 37.6 kg ± 3.9 vs. post, 37.9 kg ± 4.5, *n* = 4). Meanwhile, their average weight and fat mass significantly decreased. Furthermore, exercise performance was not influenced. In comparison, a typical western diet did not cause any significant difference in the experimental period [61]. Animal experiments have also provided evidence. An eight-week KD enhanced endurance exercise capacity in mice, while the percentage of muscle mass was not altered [37]. Kephart et al. conducted a three-month KD-intervention experiment in CrossFit trainees [62]. The authors warned that though no significance was observed in the present study, KD may reduce leg muscle mass; that is to say, prolonged KD may negatively influence muscle mass. However, combining long-term KD with other supplementation or periodic nutritional strategies may be a solution to counteract any loss of mass.

Blood levels of creatine kinase (CK) and lactate dehydrogenase (LDH) are usually used as muscle-damage markers. In Zajac’s research, a four-week KD significantly decreased CK and LDH activities, both at rest and after a 105-min cycling exercise [50]. Similar results were also found in animal studies. After a 24-h rest post-exhaustive exercise, plasma CK was reduced in KD-fed subjects, while in the normal-fed subjects, plasma CK was still significantly elevated due to the influence of acute exercise [37]. Organ damage-preventive effects were also reported. Blood urea nitrogen (BUN) and alanine transaminase (ALT) were usually employed as markers of exercise-induced acute renal damage and acute hepatic damage. In the previously narrated experiments, both markers were significantly decreased by a KD following an exhaustive exercise, both immediately after exercise and 24 h post-exercise [35,36]. 

## 5. Prospect: Combination of a KD with Other Supplementations, Exogenous Ketone Bodies, and Their Limitations

Though solid evidence has been collected about the potential of KD as a nutritional approach, based on recent studies, it is also worth addressing that a traditional KD may have limits and flaws. In a recent study, following a 12-week KD, corpuscular hemoglobin and mean corpuscular hemoglobin concentration decreased within endurance athletes (*n* = 9) [63]. Iron inefficiency and other pathological conditions may contribute to the above results, thus impairing endurance exercise capacity. Red blood cell status may need to be occasionally measured for athlete wellbeing. Vitamin E and iron supplementation or a high-altitude training plan that could promote erythropoietin production may help to resolve this problem [64,65,66].

As we discussed above, a KD may induce muscle loss and excess oxidative stress. To prevent oxidative damage, oxidative state and muscle mass may need to be occasionally monitored; antioxidant consumption is also recommended [37]. Green tea extract, curcumin, and some polyphenols have been extensively reported for their anti-oxidative properties together with endurance-enhancing properties; thus, the combination of KD and such antioxidants may be preferred [67,68,69,70,71,72,73,74,75,76,77,78,79,80,81,82,83,84]. Branched-chain amino acids (BCAA) are widely reported for their protective effects on muscle atrophy and muscle damage, and the combined use of BCAA or BCAA-like supplements and KD may be preferred to sustain muscle mass [85,86,87,88,89,90,91]. 

Another supplement that should be addressed is ketone supplements. These supplements usually constitute β-Hydroxybutyrate [92,93,94]. Exogenous ketone body ingestion could elevate blood ketones in a short time and induce acute ketosis [93,94,95,96,97]. It is reported that one-week or eight-month administration of ketone salt supplementation may be beneficial for multi-organ markers of oxidative stress and mitochondrial function [98]. However, there is insufficient data to prove whether exogenous ketone ingestion could successfully induce keto-adaptation, and the presumption itself may be speculative [99]. 

In a recent review about nutritional supplements that could induce ketosis, a negative conclusion was also reported; exogenous ketones may inhibit endogenous ketone production, and this application is more of mimicking effects [100]. 

KD was reported to cause hepatic insulin resistance in several studies, which could be attributed to increased hepatic diacylglycerol content that may lead to impaired insulin signaling. Hepatic steatosis and inflammation, as well as increased endoplasmic reticulum (ER) stress, were found in mice fed by a 12-week KD. Increased accumulation and size of macrophages were found in 12-week KD-fed mice, indicating that long-term KD application may aggravate exercise-induced inflammation. Apoptosis-related gene X-box binding protein 1 (Xbp1) was found to increase in the liver, indicating ER stress and hepatocyte apoptosis. A histological study showed that KD induced obvious fat vacuoles [101]. As the primary site for lipid metabolism, patients with impaired hepatic function, such as nonalcoholic fatty liver, should be cautious. Pancreas α-cell and β-cell masses were both reduced following a 22-week KD administration, thus leading to glucose intolerance [102]. Restricted intake of carbohydrate-enriched fruits or cereals may induce a headache, according to several KD studies [103,104]. A multi-task test that requires higher mental processing may be adversely affected by the KD according to a study [105]. These are potential symptoms that might happen during keto-adaptation and continual ketosis. A KD has potential and limitations, and further studies are warranted to investigate the combination of KD and other supplementations, or how to apply KD as a periodic nutritional approach, in order to discover a strategy for KD application.
